# Microbe-associated molecular pattern-induced calcium signaling requires the receptor-like cytoplasmic kinases, PBL1 and BIK1

**DOI:** 10.1186/s12870-014-0374-4

**Published:** 2014-12-19

**Authors:** Stefanie Ranf, Lennart Eschen-Lippold, Katja Fröhlich, Lore Westphal, Dierk Scheel, Justin Lee

**Affiliations:** Stress and Developmental Biology, Leibniz Institute of Plant Biochemistry, Weinberg 3, Halle/Saale, D-06120 Germany; Phytopathology, Center of Life and Food Sciences Weihenstephan, Technische Universität München, Emil-Ramann-Str. 2, Freising, Weihenstephan D-85350 Germany

**Keywords:** Calcium, *Changed calcium elevation* (*cce*) mutants, Receptor-like cytoplasmic kinase, Signaling

## Abstract

**Background:**

Plant perception of conserved microbe-derived or damage-derived molecules (so-called microbe- or damage-associated molecular patterns, MAMPs or DAMPs, respectively) triggers cellular signaling cascades to initiate counteracting defence responses. Using MAMP-induced rise in cellular calcium levels as one of the earliest biochemical readouts, we initiated a genetic screen for components involved in early MAMP signaling in *Arabidopsis thaliana*.

**Results:**

We characterized here the “*changed calcium elevation 5*” (*cce5*) mutant, where five allelic *cce5* mutants were isolated. They all show reduced calcium levels after elicitation with peptides representing bacteria-derived MAMPs (flg22 and elf18) and endogenous DAMP (AtPep1), but a normal response to chitin octamers. Mapping, sequencing of the mutated locus and complementation studies revealed *CCE5* to encode the receptor-like cytoplasmic kinase (RLCK), *avrPphB sensitive 1-like 1* (*PBL1*). Kinase activities of PBL1 derived from three of the *cce5* alleles are abrogated *in vivo*. Validation with T-DNA mutants revealed that, besides PBL1, another RLCK, Botrytis-induced kinase 1 (BIK1), is also required for MAMP/DAMP-induced calcium elevations.

**Conclusions:**

Hence, PBL1 and BIK1 (but not two related RLCKs, PBS1 and PBL2) are required for MAMP/DAMP-induced calcium signaling. It remains to be investigated if the many other RLCKs encoded in the Arabidopsis genome affect early calcium signal transduction – perhaps in dependence on the type of MAMP/DAMP ligands. A future challenge would be to identify the substrates of these various RLCKs, in order to elucidate their signaling role between the receptor complexes at the plasma membrane and downstream cellular signaling components.

**Electronic supplementary material:**

The online version of this article (doi:10.1186/s12870-014-0374-4) contains supplementary material, which is available to authorized users.

## Background

During their infection attempt, microbes activate intracellular signaling cascades in their potential host. Specific pattern-recognition receptors (PRRs) from the host recognize conserved microbe-associated molecular patterns (MAMPs) or certain signature molecules resulting from tissue damage, often designated as damage-associated molecular patterns (DAMPs) [1]. PRRs are typically receptor-like kinases (RLKs), such as FLS2 (Flagellin-Sensing 2), EFR (Elongation Factor Tu Receptor) or PEPR1/PEPR2 (AtPep-Receptor 1/2). These recognize the MAMPs, flg22 (N-terminal flagellin-derived peptide), elf18 (N-terminal fragment of Elongation Factor Tu) and the DAMP, AtPep1, respectively [2]. Upon binding of the respective ligand [3-5], FLS2, PEPR1/PEPR2 or EFR hetero-oligomerize with BAK1 (BRI1-Associated Kinase 1), a kinase originally found as an interactor of the brassinosteroid hormone receptor, BRI1 [6]. Recent structural studies indicate that BAK1 is also in direct contact with the C-terminal part of the FLS2-bound flg22, and may thus be considered a co-receptor [7]. Accordingly, *bak1* mutants are impaired in responses to these MAMPs/DAMPs [5,8,9]. Thus, BAK1 acts as protein partner (or co-receptor?) for multiple pathways in plant immunity and development [10]. On the other hand, signaling induced by other MAMPs, such as chitin is independent of BAK1 [11]. This difference may be a consequence of the different structure of the potential receptor(s) required for perceiving chitin, CERK1 (Chitin Elicitor Receptor Kinase 1), a LysM-containing RLK in Arabidopsis [12-14] as compared to the LRR-type RLKs such as FLS2, EFR or PEPR1/R2.

Among the earliest signaling events after MAMP/DAMP perception are ion fluxes across the plasma membrane including influx of calcium into the cytosol [8,15-17]. The elevation of cytosolic calcium is detected by a number of calcium-binding “decoder” proteins such as calmodulins or calcium-dependent protein kinases (CPKs) or Calcineurin B-like (CBL) proteins and their partners, CBL-interacting protein kinases (CIPKs) to further transmit the signal [18,19]. Calcium, as a signaling molecule, is a prerequisite for most downstream responses elicited by MAMPs/DAMPs. For instance, production of reactive oxygen species (ROS) by the NADPH oxidase, RBOHD, in Arabidopsis [20] is a calcium-dependent process stimulated by direct binding of calcium to EF-hands in the N-terminus of RBOHD. Furthermore, the calcium-dependent protein kinase 5 (CPK5) phosphorylates RBOHD to promote its activity [21]. Activation of mitogen-activated protein kinases (MAPKs) also requires calcium since depletion of extracellular calcium or inhibition of calcium channels block MAMP-induced MAPK activation [9,22].

The importance of calcium for plant immunity is also indirectly supported by the observation that phytopathogenic bacteria secrete extracellular polysaccharides to sequester apoplastic calcium and attenuate host MAMP signaling [23]. However, much of plant calcium signaling remains to be discovered, in particular, the steps between perception of MAMPs/DAMPs and generation of the calcium signals. We used an apoaequorin-expressing transgenic *Arabidopsis thaliana* line to investigate MAMP signaling events in whole seedlings [9]. Aequorin is a calcium sensitive reporter for measuring changes in cellular calcium levels [24]. Upon binding calcium, it oxidizes the bound coelenterazine prosthetic group into excited coelenteramide, which emits blue light at 469 nm. The so-called L/L_max_ ratio of the aequorin-generated luminescence (L) to the total remaining aequorin (L_max_) is used as an estimate of relative calcium levels. With the appropriate calibration parameters, it is also possible to convert the L/L_max_ values into absolute cytosolic calcium concentrations [25].

We previously demonstrated that the aequorin-based measurement is amenable to high throughput screening and used it to isolate mutants with a “*changed calcium elevation* (*cce*)” phenotype after flg22 elicitation. The first sets of identified *cce* mutants were the FLS2 receptor and its partner kinase, BAK1 [26]. These mutants represent proof-of-principle of the suitability of the screen in finding signaling components between ligand recognition and calcium flux. This current work reports the characterization of the *cce5* mutant and the identification of the receptor-like cytoplasmic kinase (RLCK), *PBS1-like 1* (*PBL1*) being the *CCE5* gene, where PBS1 stands for avrPphB sensitive 1, an RLCK targeted by the *Pseudomonas syringae* pv. phaseolicola protease avrPphB [27]. The analysis of mutants of three related RLCKs revealed an additional requirement of Botrytis-induced kinase 1 (BIK1) for the MAMP/DAMP-induced calcium elevation.

## Results

### The *changed calcium elevation 5* (*cce5*) mutant is affected in early signaling

**Figure 1:**
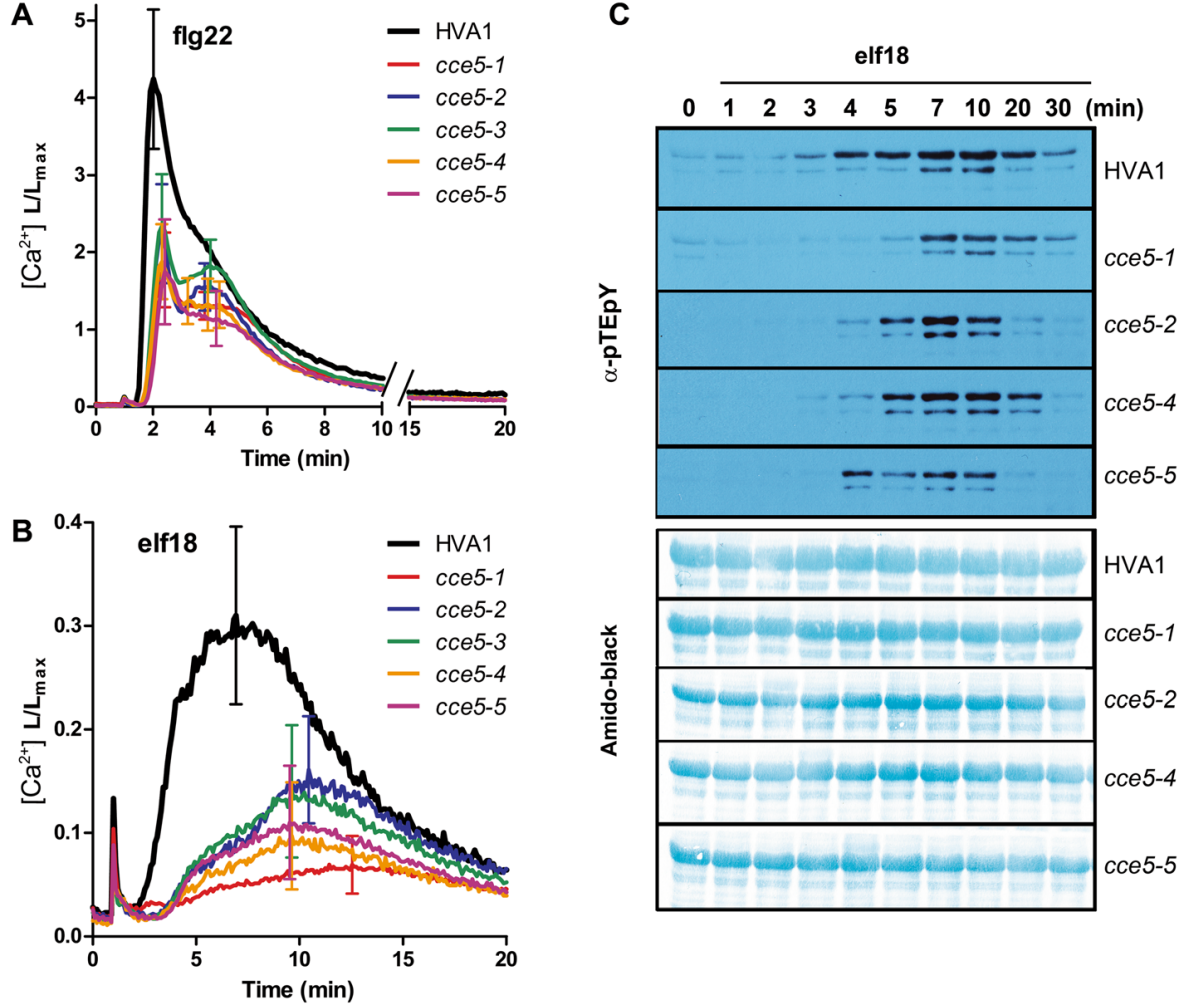
**Early responses are reduced in the** ***changed calcium elevation 5*** **(** ***cce5*** **) mutants compared to the parental HVA1 line.** Seedlings (~8 days old) were elicited with 1 μM of flg22 **(A)** or 1 μM elf18 **(B)** and the calcium levels measured. Relative calcium levels are depicted as L/Lmax ratio (where L/Lmax = luminescence counts per sec/total luminescence counts remaining). Error bars represent standard deviation (n > 12 seedlings). MAPK activation is shown by immunoblotting (α-pTEpY) for the phosphorylated MAPKs after 1 μM elf18 elicitation **(C)**. Amido black staining of the nitrocellulose membrane was used to estimate equal loading. For MAPK activation after flg22 treatment, see Additional file 1: Figure S1.

Four other independently isolated *changed calcium elevation* (*cce*) mutants did not restore a “normal” calcium response to flg22 in the F1 generation when crossed to the previously described *cce5* mutant [26] (data not shown). The lack of complementation suggests that these five *cce* mutants are allelic and thus designated as *cce5-1* to *cce5-5*. All five *cce5* mutant lines show a reduced flg22- and elf18-induced calcium rise compared to the parental HVA1 line; however, the reduction in the elf18-induced calcium levels appears to be stronger than with flg22 (Figure 1A, B). Correspondingly, elf18-induced MAPK activation was partially reduced and delayed (Figure 1C). Surprisingly, the reduction in flg22-induced MAPK activation was not as obvious as for elf18. It was only visible if a lower concentration (e.g. 10 nM) of the flg22 peptide was used; at higher concentrations, no difference in comparison to HVA1 was discernible (Additional file 1: Figure S1). Thus, CCE5 may have different signaling role(s) for these two MAMPs. Similarly, other rapid responses such as reactive oxygen species (ROS) accumulation was also reduced for the *cce5* alleles, when treated with flg22 or elf18 (Additional file 1: Figure S2). Since MAPK activation and ROS accumulation occur within minutes upon elicitation, *cce5* is “mutated” in some early signaling component(s).

### Differential MAMP/DAMP response of the *cce5* mutants is reminiscent of a BAK1-dependent response, but *CCE5* is not *BAK1*

**Figure 2:**
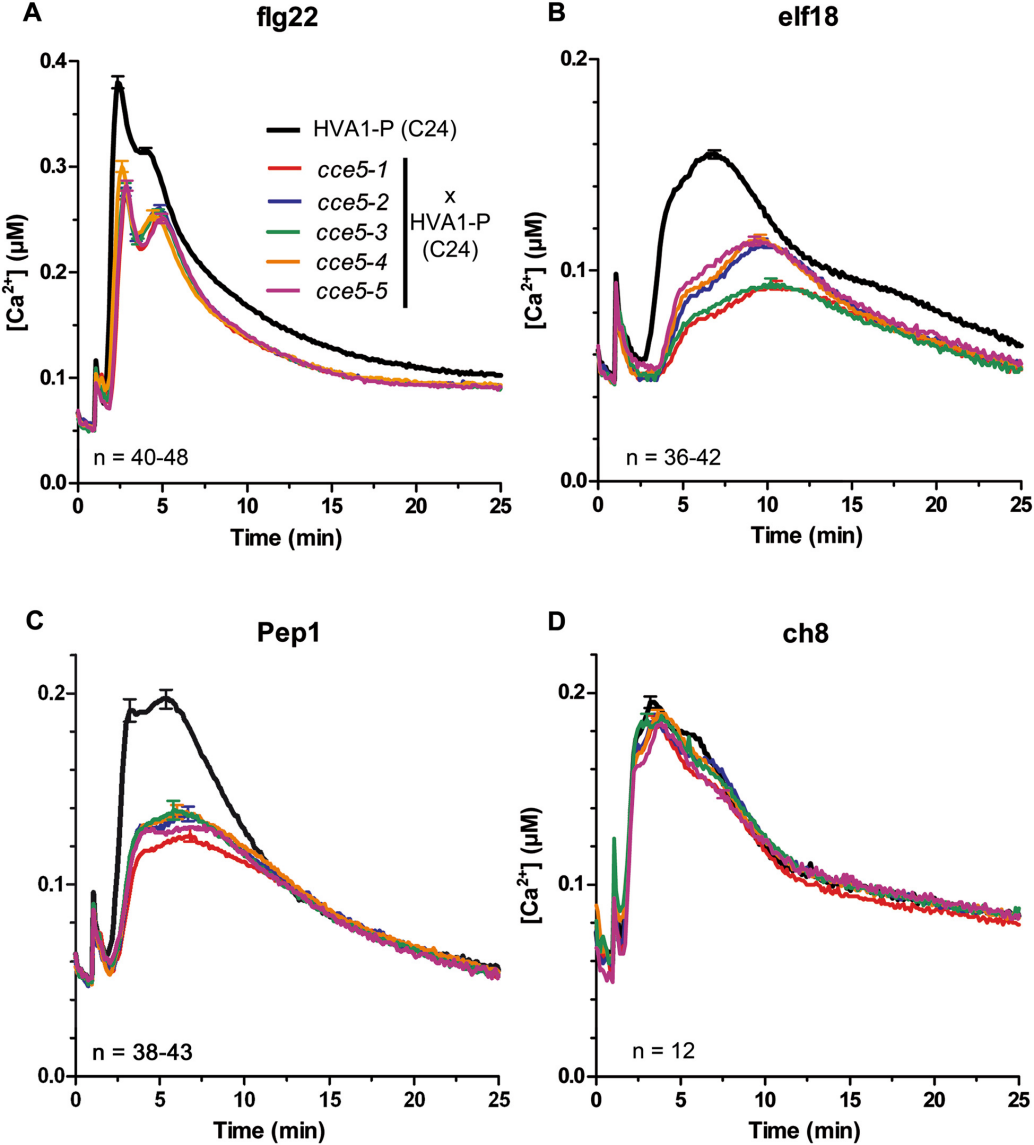
**Calcium elevation is reduced after treatment with flg22 (A), elf18 (B) and AtPep1 (C) but not with chitin octamer (ch8) (D) in all five** ***cce5*** **mutants.** To enable absolute cytosolic calcium measurements, the parental HVA1 (C24) line was transformed with the pMAQ2 construct (designated as HVA1-P) and used as a reference. The indicated genotypes were crossed with HVA1-P (i.e. a C24 Arabidopsis accession expressing cytosolic apoaequorin). Seedlings from the F2 populations were screened for the *cce* phenotype to identify homozygous *cce5* plants, the mutations verified by CAPS marker analysis, and the progenies used for calcium measurements. Error bars represent standard errors of the mean.

The background line for the *cce5* mutants carries the so-called “*HVA1*” transgene (in the *Arabidopsis thaliana* C24 ecotype), where the aequorin reporter is targeted to the tonoplast outer surface, which detects calcium exiting the vacuole but does not permit determination of absolute calcium concentrations [28]. In order to perform calibrations required for calculating absolute calcium concentrations, and to also confirm the effect of the *cce5* mutation on cytosolic calcium levels, the five *cce5* alleles were crossed into a HVA1 line (HVA1-P) that was additionally transformed with a cytosolic apoaequorin (pMAQ2) construct. These “back-crossed” lines also reduce possible effects from secondary mutations arising from the chemical mutagenesis. Seedlings from the F2 populations were screened for the reduced MAMP-induced calcium phenotype to identify homozygous *cce5* plants and the mutations were verified by CAPS marker analysis (see Additional file 1: Table S2). Using these lines, a survey of different MAMPs/DAMPs showed reduced calcium responses to flg22, elf18 and AtPep1 but a normal response to chitin octamers (ch8) in *cce5* (Figure 2). This differential phenotype to various MAMP/DAMPs is reminiscent of a “BAK1-dependent” type of response, where BAK1 is not required for the calcium elevation induced with ch8 [9].

**Figure 3:**
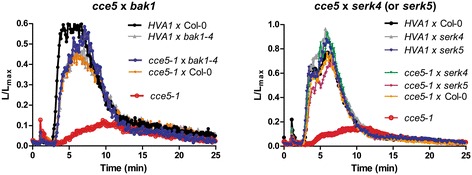
***CCE5*** **is not allelic to** ***BAK1*** **,** ***SERK4*** **or** ***SERK5.*** The *cce5-1* allele was crossed to *bak1-4* (left panel) or to *serk4* or *serk5* mutants (right panel). Crosses with Col-0 or the HVA1 parent were used as controls. Relative calcium (L/Lmax) levels were determined in the F1 crosses after elf18 (1 μM) treatment – showing that the *cce5* phenotype was complemented when crossed with the *bak1*, *serk4* and *serk5* mutants and therefore not allelic to these genes. (n = 8–14 seedlings)

BAK1 is the BRI1-associated receptor kinase shown to interact with FLS2, EFR and PEPR1/R2 receptors in a ligand-dependent manner [29]. To exclude that the *cce5* mutants are weak alleles of *BAK1* or the related *SOMATIC EMBRYOGENESIS RELATED KINASE* (*SERK*) members, *cce5-1* was crossed to the mutants *bak1-4, serk4-1* and *serk5-1* [5]. Since the *cce5* effect was most prominent for elf18 elicitation, we measured elf18-induced calcium fluxes in F1 seedlings, and observed that the *cce5* phenotype was complemented (Figure 3). This result indicates that *CCE5* is not allelic to *BAK1, SERK4* or *SERK5* and hence *cce5* is mutated in a different gene.

### *CCE5* encodes the receptor-like kinase, PBL1

**Figure 4:**
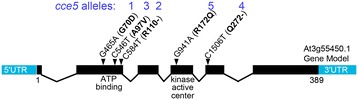
**Scheme of** ***PBL1*** **(At3g55450) gene structure and mutations in the** ***cce5*** **alleles.** Genomic DNA was prepared from the five *cce5* mutants and the *PBL1* gene amplified by PCR and sequenced. The detected single nucleotide polymorphisms (SNPs) and the resulting amino acid exchanges are indicated above the exons. The gene model *At3g55450.1* encoding a protein of 389 amino acids is used to designate the position of the amino acid exchanges. Locations of key kinase domains (such as ATP binding site and the kinase active center), relative to the corresponding mutations, are marked.

To identify the *CCE5* gene, an F2 population was generated by crossing *cce5-1* with the Arabidopsis accession L*er*-0. Segregation analysis with 36 F2 plants indicated that *CCE5* is linked to the aequorin transgene, and located on chromosome 3 between the INDEL markers CER460928 (1 recombinant) and 473892 (1 recombinant) [30]. The map positions of CER460928 and 473892 are 17.243303 and 21.186345 Mbp (based on TAIR 10). This interval comprises 1107 gene loci, including the *PBS1-like 1* gene (*PBL1*, At3g55450) that encodes a receptor-like cytoplasmic kinase (RLCK). Sequencing of the *PBL1* gene of the *cce5* mutants revealed single nucleotide polymorphisms (SNP) in all five *cce5* alleles, but not in the *PBL1* sequences from two other *cce* mutants, *cce7* and *cce8* [26]. These SNPs lead to two premature stops (*cce5-2*/R110- and *cce5-4*/Q272-) and three amino acid exchanges (*cce5-1*/G70D, *cce5-3*/A97V and *cce5-5*/R172Q) in the PBL1 sequence (Figure 4). Two gene models are predicted for *PBL1* transcripts in the TAIR database, but since we could not detect transcripts for the predicted alternatively spliced gene model At3g55450.2 (data not shown), we used the 389 amino acid long PBL1 protein (predicted by the gene model At3g55450.1) to designate the positions of the amino acid exchanges in the *cce5* mutated proteins.

PBL1 or PBS1-like 1 belongs to subfamily VII of RLCKs (Additional file 1: Figure S3) that include the founding member avrPphB-susceptible 1 (PBS1) [31] and the Botrytis-induced kinase 1 (BIK1) [32,33]. To further validate that *CCE5* is *PBL1*, a T-DNA insertion mutant of *PBL1* was isolated. For comparison, T-DNA mutants of related members of this family of RLCKs shown to be involved in PTI (BIK1, PBS1, PBL2) [34], were also obtained. The T-DNA mutants were crossed with the cytosolic aequorin-expressing (pMAQ2 in Col-0 background) transgenic line. However, silencing of the aequorin reporter was observed in some crosses, and in these cases (i.e. for *pbl2* and *pbs1*), an independently generated line with the apoaequorin expression driven by the *UBIQUITIN10* promoter (*pUBQ-AEQ* in Col-0 background) was used for crossing.

**Figure 5:**
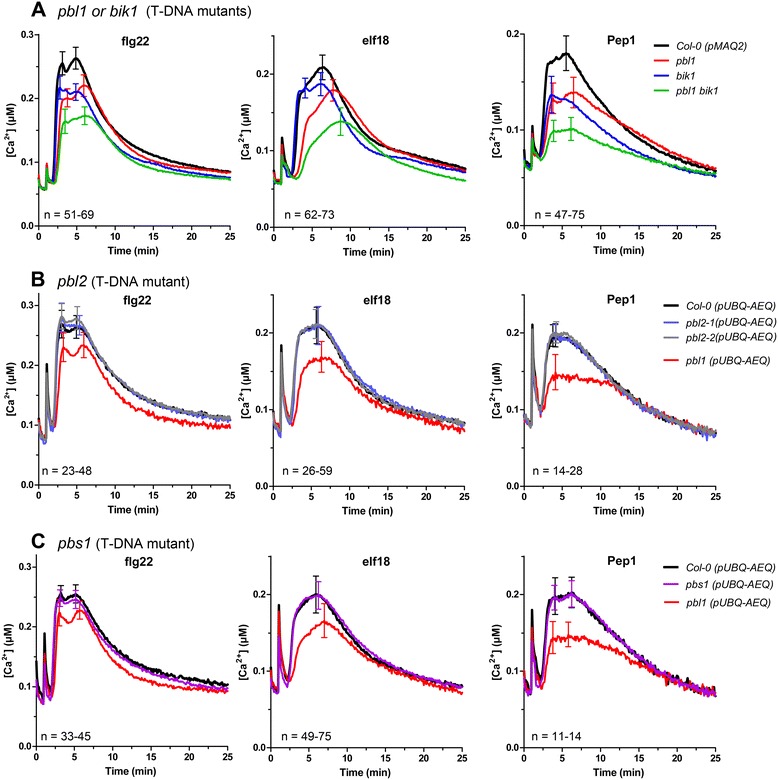
**MAMP/DAMP-induced calcium elevations in T-DNA insertion mutants of** ***PBL1*** **,** ***BIK1*** **,** ***PBL2*** **and** ***PBS1*** **.** T-DNA insertion mutants of *pbl1*, *bik1* or a *pbl1bik1* double mutant were crossed with the pMAQ2 aequorin transgenic line. After verifying the mutant genotypes by PCR, changes in calcium levels induced by the indicated MAMPs were measured in the F2 or F3 generations **(A)**. Due to silencing when crossed to the pMAQ2 reporter for the *pbl2* **(B)** and *pbs1* **(C)** mutants, crosses were made with an independently generated *pUBQ-AEQ* line with the expression of the apoaequorin reporter driven by the *UBIQUITIN-10* promoter. Hence, the parental *pUBQ-AEQ* line in Col-0 background was used as a reference for the wild type calcium signature for *pbl2* and *pbs1*. (Two independent *pbl2* mutant alleles were included). Error bars represent standard errors.

Consistent with the *cce5* mutants, a reduced calcium elevation induced by flg22, elf18 and AtPep1 could be recapitulated in the *pbl1* T-DNA mutant (Figure 5A). Similarly, a *bik1* T-DNA mutant was compromised in calcium elevations induced by flg22, elf18 and AtPep1 whereas *pbl2* and *pbs1* showed no reduction in calcium elevation (Figure 5B,C). In the experiments with *pbl2* and *pbs1*, a *pbl1* line crossed with the *pUBQ-AEQ* line was used as a control to demonstrate that the lack of phenotype in *pbl2* and *pbs1* is not due to a different aequorin reporter background. Additionally, a *pbl1bik1* double mutant showed further reduction of the flg22-, elf18- or AtPep1-induced calcium elevations compared to the *pbl1* and *bik1* single mutants (Figure 5A). One should also note that the altered calcium signature differs between the *bik1* and *pbl1* mutants (Figure 5A). Taken together, members of this RLCK family contribute differentially to MAMP/DAMP-induced calcium elevation and there are partial redundancies between *PBL1* and *BIK1*.

### Differential downstream responses in the *pbl1* and/or *bik1* mutants

**Figure 6:**
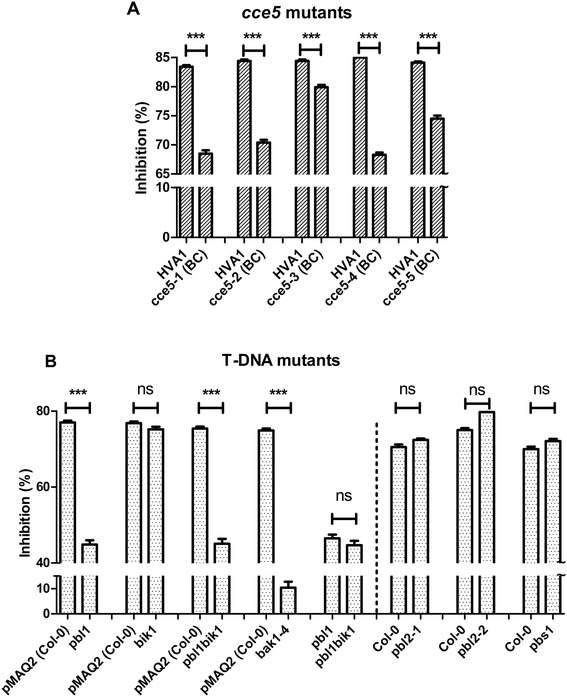
**MAMP-induced growth inhibition is dependent on** ***PBL1*** **.** All five *cce5* alleles were backcrossed (BC) with the HVA1 parent before the growth inhibition assay **(A)**. For the T-DNA mutants **(B)**, either Col-0 or the *pMAQ2* transgenic line (in Col-0 background) was used as reference. Wild type and mutant seedlings were grown on ATS plates with or without 1 μM of flg22. Root length was measured after 14 days and depicted as percent inhibition. Two-way ANOVA was used to assess significant differences in root length (*** = p < 0.001; ns = not significant).

Due to the possible trade-offs between defense and growth regulation, continuous activation of defense responses is often detrimental for plant growth. Growth inhibition assays are thus a facile measure of defense activation. This is performed by comparing root lengths of seedlings grown on normal and MAMP-containing agar plates. For this assay, we grew the two genotypes to be compared side-by-side on the same plate to eliminate differences that may arise between plates (e.g. the amount of agar per plate affects the absolute amount of MAMPs available to the seedlings). Two-way-ANOVA was used to determine the statistical significance of differences in root lengths between the genotypes and the treatments, respectively. For simplicity, percent inhibition (as compared to the average root length of plants grown on standard plates) is shown in Figure 6. To reduce the effects of secondary mutations, all *cce5* mutants were backcrossed to the HVA1 parent, screened for the *cce* phenotype and confirmed by CAPS marker analysis before the assay. Reduced flg22-mediated growth inhibition compared to the corresponding background lines could be seen for all five backcrossed *cce5* mutants (Figure 6A) and the *pbl1* T-DNA mutant, but not for *pbs1* and *pbl2* (Figure 6B). Surprisingly, despite the reduced calcium increase (Figure 5A), the *bik1* mutant showed no reduction in root growth inhibition. There was also no additive growth reduction in the *pbl1bik1*double mutant (Figure 6B). A direct comparison between the *pbl1* single mutant and the *pbl1bik1*double mutant assayed on the same plate also showed no statistically significant difference in root growth inhibition (Figure 6B). Since *PBL1* and *BIK1* expression levels in roots are quite similar (i.e. similar signal intensities throughout the currently available microarray experiments, as analyzed by Genevestigator), the differential impact on flg22-mediated growth reduction is not due to lack of *BIK1* expression in roots. Thus, while *PBL1* and *BIK1* have an impact on early signaling events like calcium increase, *PBL1* plays a more important role than *BIK1* in the late root growth inhibition response to flg22. On the other hand, BIK1, but not PBL1, has been shown to play an important role in flg22-mediated resistance to subsequent *Pseudomonas syringae* infection, while both BIK1 and PBL1 regulate callose deposition induced by selected MAMPs and defense gene expression [34]. Hence, PBL1 and BIK1 have overlapping but also distinct roles in defense signaling/responses as is also reflected by the wildtype-like phenotype of *pbl1* plants compared to the altered growth phenotype and the constitutive SA accumulation of *bik1* mutants [33].

**Figure 7:**
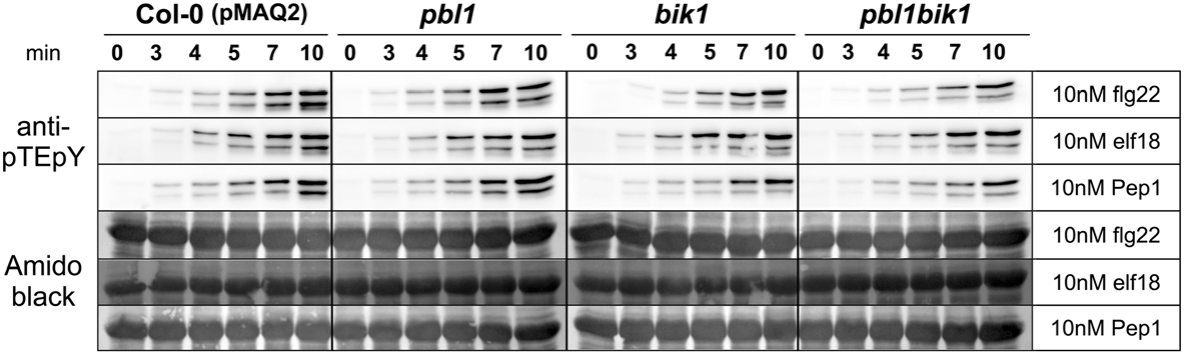
**MAPK activation profile in the T-DNA insertion** ***pbl1*** **,** ***bik1*** **or** ***pbl1bik1*** **double mutants.** 14-day-old liquid-grown seedlings were equilibrated in 1.5 ml of fresh MS medium for ~24 h, and elicited by adding 0.5 ml of media containing a 4-fold concentrated stock of the indicated MAMP/DAMPs. Samples were collected at the indicated time points (min) after treatment and proteins were extracted for immunoblotting to detect phosphorylated (i.e. activated) forms of the MAPKs. Amido black staining of the nitrocellulose membranes was used to estimate equal loading.

Since calcium acts upstream of MAPK activation [9,22], we analyzed MAPK activation in the T-DNA mutants of *PBL1*, *BIK1* and the double mutant. However, there was no reduction in MAPK activation by flg22, elf18 and Pep1 in *pbl1*, *bik1* and *pbl1bik1* compared to their Col-0 (pMAQ2) background line (Figure 7). This is in contrast to the reduced elf18-induced MAPK activation (Figure 1C) and the dose dependent reduction in flg22-induced MAPK activation (Additional file 1: Figure S1) in the *cce5* mutants. Since the reduction of MAPK activation could be seen in multiple *cce5* lines, the difference is unlikely to be due to secondary mutations in the ems-mutagenized lines.

### Kinase activities and proper localization of RLCKs determine downstream signaling

**Figure 8:**
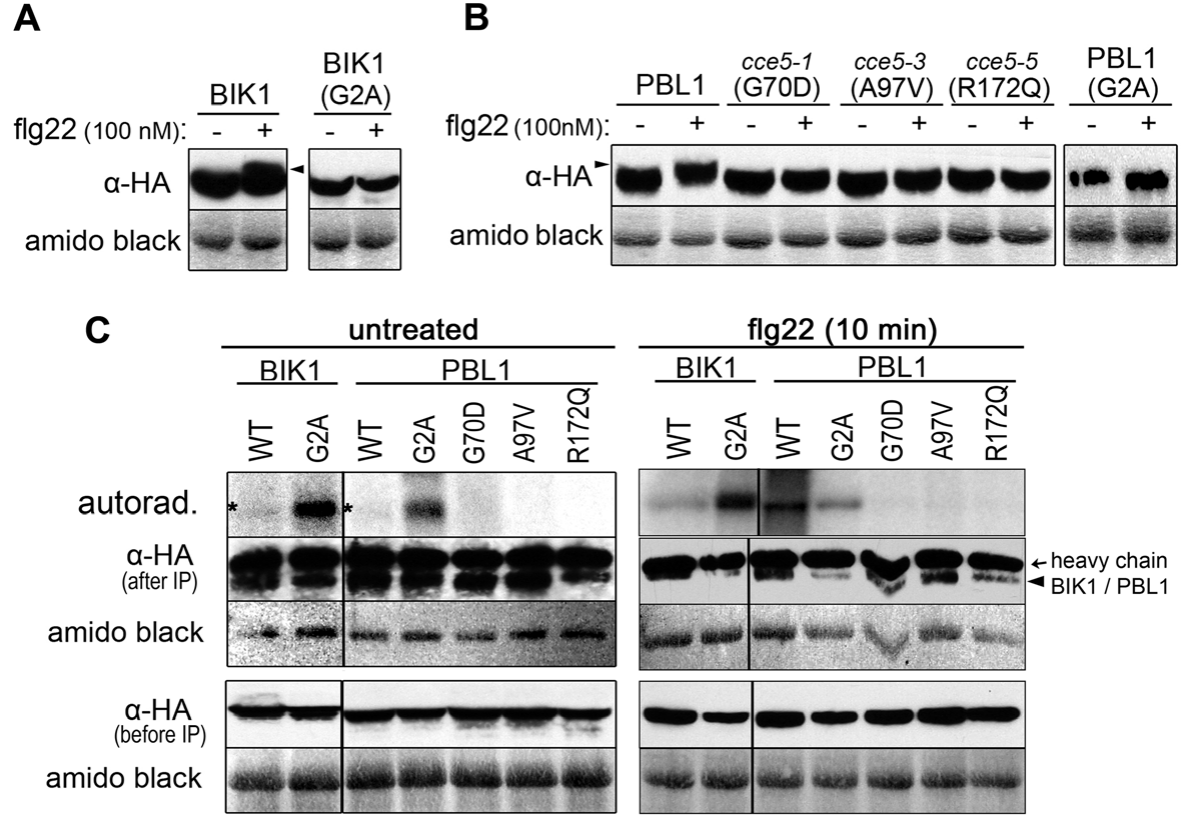
***In vivo*** **phosphorylation of PBL1 or BIK1 after flg22 treatment.** Protoplasts were transfected with plasmids expressing HA-tagged BIK1 **(A)**, PBL1 **(B)**, or the indicated variants. After overnight expression of the proteins, the protoplasts were treated with 100 nM flg22 (10 min), harvested and subjected to western blotting with anti-HA. *In vivo* phosphorylation is implicated by a reduced mobility of the protein (highlighted with black arrowheads). Amido black staining of the nitrocellulose membranes was used to estimate equal loading. In **(C)**, autophosphorylation of the immunoprecipitated kinases was used to determine if the kinase activities have been compromised by the mutations. The experiment was performed three times with similar outcome. Note that the autophosphorylation of the wild type (WT) kinases in the untreated protoplasts is variable and typically low but a weak band can be seen (indicated by asterisks). Autophosphorylation of the G70D, A97V and R172Q variants were always not visible (or lower than the wild type kinases).

After MAMP stimulation of plants, a reduced mobility of PBL1 and BIK1 protein bands in polyacrylamide gels (i.e. a “mobility shift”), indicative of *in vivo* phosphorylation of the kinases, has been reported [34,35]. Since three of the *cce5* alleles are predicted to encode PBL1 proteins with a single amino acid exchange (Figure 4), we tested these mutated PBL1 proteins as well as BIK1 with regard to the gel mobility shift. As a negative control, we mutated the presumed myristoylation site (G2A) of PBL1 and BIK1, which is expected to prevent the proteins from targeting to the plasma membrane. All these constructs were tagged with a C-terminal HA epitope for western blot detection and transiently expressed in Arabidopsis mesophyll protoplasts. A mobility shift could be seen for wild type PBL1 and BIK1 after flg22 treatment of the protoplasts, but not for the G2A myristoylation site variants and the G70D, A97V and R172Q PBL1 variants (Figure 8A, B). This indicates that there is no *in vivo* phosphorylation of the mutated protein variants after flg22 treatment.

To test if the kinase activities have been affected, we immunoprecipitated the proteins with anti-HA antibodies and incubated the immunoprecipitates in the presence of radioactive ATP to enable autophosphorylation. After separation on a SDS-polyacrylamide gel, radioactive signals corresponding to the proteins could be seen for the wild type and the G2A mutated PBL1 and BIK1, suggesting that these are still active kinases (Figure 8C). Notably, the wild type BIK1 or PBL1 autophosphorylation signals are weak before flg22 treatment (highlighted with asterisks in Figure 8C, left panel). However, compared to the wild type PBL1 protein, there was no (or strongly reduced) autophosphosphorylation of the G70D, A97V or R172Q mutated PBL1 variants (Figure 8C). Taken together, these three *cce5* mutations led to the loss of PBL1 kinase activity, while the mis-localization of PBL1^G2A^ and BIK1^G2A^ proteins prevented the *in vivo* phosphorylation of these kinases after flg22 signaling.

**Figure 9:**
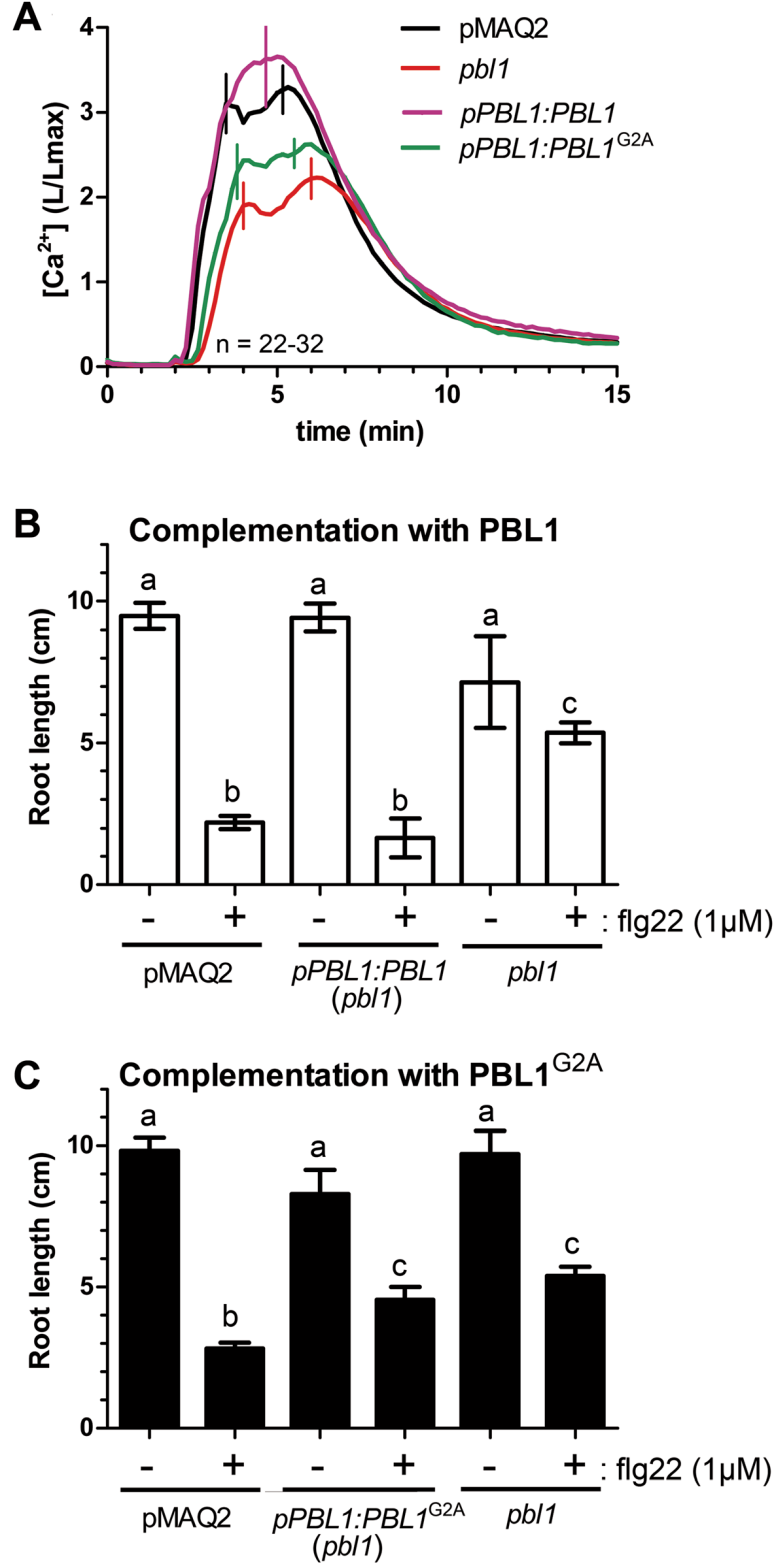
**Complementation of** ***pbl1*** **T-DNA mutant.** The T-DNA *pbl1* mutant was transformed with a genomic fragment encompassing the *PBL1* locus. As a negative control, mutation of the myristoylation site (G2A) prevented complementation in the flg22-induced calcium elevation phenotype **(A)** and the flg22-mediated growth arrest **(B, C)**. In **(A)**, the calcium levels of the *pPBL1:PBL* ^*G2A*^-complemented seedlings (green trace) are not statistically significant from calcium levels in the *pbl1* mutant (red trace). In **(B)** and **(C)**, the letters above each bar show the statistical significance groupings based on two-way ANOVA. (n = 14–27 seedlings).

As a final proof that PBL1 is required for the MAMP-induced calcium elevation, we introduced a genomic DNA fragment encompassing the *PBL1* gene locus into the *pbl1* T-DNA mutant. This genomic fragment complemented the reduced calcium elevation (Figure 9A) and the root growth inhibition (Figure 9B) phenotype in *pbl1*. As a negative control, mutation of the putative myristoylation site (G2A) prevented the complementation of the *cce* phenotype (Figure 9A) as well as the flg22-induced root growth inhibition (Figure 9C). Hence, myristoylation and proper targeting of PBL1 to plasma membrane is essential for signaling function of PBL1.

## Discussion

### Specific RLCKs required for calcium signaling and MAMP/DAMP signaling

Using both forward and reverse genetics as well as complementation studies, we identified the *CCE5* gene as encoding the receptor-like cytoplasmic kinase, PBL1, and with the *cce5* mutants, we isolated five new *pbl1* alleles. We further showed that another RLCK, BIK1, but not PBS1 and PBL2, is required for the MAMP/DAMP-induced calcium elevation pathway. This is in agreement with a recent report that flg22-induced calcium flux is compromised in *bik1* [36]. Using these new *pbl1* alleles and T-DNA insertion mutants, a MAMP-mediated root growth inhibition assay confirmed the requirement of *PBL1* for downstream signaling leading to growth arrest. However, the *bik1* mutation had no apparent effect on flg22-mediated growth arrest. Thus, despite both *pbl1* and *bik1* mutants showing a reduced MAMP/DAMP-induced calcium elevation, downstream growth arrest effects differ. One explanation is that PBL1 and BIK1 are not simply redundant but also have distinct signaling roles, which is reinforced by other studies showing both overlapping and distinct requirements for PBL1 and BIK1 in MAMP-induced ROS production, callose deposition, gene expression and pathogen resistance [33,34,37,38]. This notion is, in fact, evident from the different calcium signatures in the *pbl1* and *bik1* mutants (see Figure 5A). A second possibility is that a signaling critical threshold of cytoplasmic calcium level is not crucial or causal for determining the degree of growth inhibition (or other MAMP-induced responses), which would imply a more important signaling role of PBL1, compared to BIK1, in mediating the possible trade-offs between growth and defense activation. It is possible that the inverse roles of BIK1 as a positive regulator of defense but a negative regulator of brassinosteroid signaling [39] contribute to this growth differences. Recently, another RLCK, PBL27, was found to be the preferential substrate (as compared to BIK1) of the CERK1 chitin receptor. By contrast, BAK1, which is already known to phosphorylate BIK1, hardly phosphorylated PBL27. PBL27 also appears to be non-essential for flg22 signaling [40]. Thus, depending on the ligand, different RLKs or RLCKs are recruited for signaling. These findings support the distinction of BAK1 requirement for flg22, elf18 and AtPep1 signaling to that of chitin [5,9]. Our data on PBL1 requirement for optimal calcium signaling induced by flg22, elf18 and AtPep1, but not chitin (Figure 5), fits into this pattern. In conclusion, there appears to be a differential requirement for members of the RLCK family downstream of the receptors for distinct MAMP/DAMP signaling.

### Phosphorylation is essential for signal relay

The recruitment (and/or exchange) of various RLKs and RLCKs at the plasma membrane after MAMP/DAMP perception is indicative of the roles of phosphorylation cascades in early signaling. Prior to stimulation, FLS2 and BIK1 are already in a protein complex [38] and BAK1 appears to be also in complex with BAK1-interacting RLKs (BIRs) [41]. Within minutes after flg22 stimulation, FLS2 recruits BAK1 [3] to phosphorylate BIK1. Activated BIK1, in turn, cross-phosphorylates FLS2 and BAK1 [38]. BAK1 also cross-phosphorylates FLS2 but apparently at different residues as BIK1 [42]. Based on the autophosphorylation assay (Figure 8C), the kinase activities of BIK1 and PBL1 appear to be higher in the flg22-treated protoplasts. The lower activities of BIK1/PBL1 in the untreated protoplasts may imply repression by some other components (eg. phosphatases) prior to elicitation. Along this idea, it is noteworthy that the N-terminal myristoylation BIK1^G2A^ and PBL1^G2A^ mutants are routinely recovered with higher autophosphorylation levels. One may speculate that mis-localization of PBL1^G2A^ and BIK1^G2A^ prevent contact with phosphatases that are presumably present in the FLS2-BIK1 (or PBL1) protein complex to restrict defense signaling. Indeed, Ser/Thr protein phosphatase type 2A (PP2A) has been shown to associate with BAK1 and control the activation of PRR complexes [43]; and whether the same or similar PP2As negatively regulate BIK1 or PBL1 remains to be demonstrated. Recently, it was shown that both BAK1 and BIK1 are dual-specific kinases that modify both serine/threonine as well as tyrosine residues [44]. The complex series of phosphorylation between PRRs, BAK1, BIK1 and PBL1 are important as mutations abrogating activities of any of these kinases block signaling. As shown by mobility shifts in gel electrophoresis, the PBL1 protein, encoded by the *CCE5* gene, is apparently phosphorylated *in vivo* after MAMP elicitation [34]. The loss (or reduction) of the kinase activities of the *cce5-*derived protein variants reported here (Figure 8C) corresponds to changes of important residues of the kinase domain. The G70D and A97V mutations are found in the ATP binding region (i.e. the kinase subdomains I and II, respectively) while R172Q is N-terminal to the active center within kinase subdomain VI (*c.f.* Figure 4 and Figure 8C). Taken together with data from literature, the reduced calcium signaling of our newly isolated *cce5*/*pbl1* alleles shows that kinase activities of PBL1 (and all the other recruited RLKS/RLCKs) are vital for early MAMP/DAMP signaling.

### Is downstream MAPK activation affected in the *pbl1* and *bik1* mutants?

Downstream of PBL1/BIK1 phosphorylation is calcium elevation, which, in turn, has been shown through pharmacological inhibitor studies to be required for downstream MAPK activation [22]. However, despite an attenuation of calcium elevation, there was no reduction in MAPK activation by flg22, elf18 or AtPep1 in the *pbl1* and *bik1* T-DNA mutants as well as *pbl1bik1* double mutant when compared to their Col-0 (pMAQ2) background line (Figure 7). This observation is in agreement with previous reports [34,45,46]. On the other hand, we observed a reduction/delay in elf18-induced MAPK activation in the *cce5* mutants (Figure 1C), while for flg22, a weak effect could be observed when a lower concentration of the flg22 peptide was used. A possible explanation for this discrepancy may be that the mutated or truncated CCE5 proteins (with inhibitory properties) are expressed by the *cce5* alleles as opposed to the (presumably) lack of proteins in the T-DNA insertion mutants. Alternatively, the different genetic background of the mutants may also play a role. In all studies where no difference in MAPK activation was observed, the mutations were in Col-0, while the *cce5* (*pbl1*) mutants have a C24 background. We previously reported that the C24 accession has higher levels of FLS2 receptor while, on the basis of public gene expression profiling data, the opposite is true for the EFR receptor [26]. Several SNPs were also detected within the *FLS2* and *EFR* genes of HVA1 (C24) [26], which may further contribute to the different sensitivities to flg22 and elf18. Together, this may explain the observed reduced elf18-induced MAPK activation in the *cce5* mutants but only a dose-dependent flg22-induced MAPK response. Along this notion, Zhang et al. [34] also reported that callose deposition in the *pbl1* mutant was normal upon flg22 treatment but reduced when treated with elf18 [34]. Thus, the differential MAMP receptor levels and/or yet unknown alterations in other signaling components between genotypes may determine the sensitivity of the system in signal relay from PBL1 to the MAPKs and other downstream events. Additionally, the RLCK, RIPK, phosphorylates RIN4 and in analogy to the recognition of the RIN4 phosphorylation by the RPM1 resistance protein as compared to recognition of RIN4 cleavage by RPS2 [47], one could speculate that the MAMP-induced RLCK phosphorylation may also be differentially recognized by the differing configuration of resistance protein spectrum between Arabidopsis accessions.

### Localization to the membrane is a prerequisite for PBL1 and BIK1 function

Besides its kinase activities, localization of PBL1 and BIK1 appears to be important for complementation of the *cce* phenotype. BIK1, as a GFP fusion protein, has been shown to be plasma membrane-localized using heterologous expression in onion epidermal cells [33]. However, to our knowledge, it has never been experimentally determined whether this is due to targeting or recruitment by other proteins. CASTAWAY, an RLCK required for organ abscission, showed reduced plasma membrane localization when a G2A mutation was introduced for the putative myristoylation site. Myristoylation is often associated with palmitoylation to enhance membrane interactions. However, no further decrease of CASTAWAY localization was observed even when the neighboring palmitoylation site (C4S) was additionally mutated [48]. Myristoylation is thought to provide the initial but weak interaction with the plasma membrane; stabilization of this membrane localization may be further strengthened through other modifications or interactions with resident plasma membrane-localized components [49]. We now show that the G2A mutation of the putative myristoylation site of PBL1/BIK1 prevented signaling (i.e. no *in vivo* phosphorylation after MAMP treatment, Figure 8) although kinase (autophosphorylation) function is apparently intact. Furthermore, the G2A variant did not complement the *pbl1* mutation (Figure 9). In a similar manner, despite being cleaved by the avrPphB cysteine protease, the “active” PBS1 fragment that is recognized must be retained at the plasma membrane for RPS5 activation [50]. In this case, plasma membrane targeting is mediated by S-acylation of a cysteine residue in the N-terminus of PBS1. Thus, not only the function of the PBS1 protein but also the recognition of its perturbation (i.e. ETI) requires correct membrane localization. Taken together, localization of the RLCKs to the proper cell compartment is crucial for function.

### Disabling RLCK functions during pathogenesis blocks defense signaling

PBS1 is the founding member of the PBL (PBS1-like) group. The *Pseudomonas* avrPphB effector, a bacterial virulence protein injected into host cells, cleaves PBS1 via its cysteine protease activity [31]. Subsequently, it was discovered that avrPphB can cleave at least 10 other PBS1-like RLCKs [34]. Their cleavage/removal represents a virulence function of avrPphB and suggests that these PBLs/RLCKs act in resistance mechanisms against bacteria. Support for this notion is provided by studies involving the *Xanthomonas* XopAC effector, which appears to target multiple RLCKs [37]. Unlike avrPphB, XopAC does not cleave but uridylates BIK1 and RIPK in susceptible plants [45]. This transfer of uridine 5′-monophosphate to conserved phosphorylation sites in the activation loop of BIK1 and RIPK, prevents phosphorylation, thereby reducing their kinase activities and consequently inhibiting downstream defense signaling. In accordance to the arms-race hypothesis, XopAC appears to be a major avirulence factor for recognition in resistant plants such as the Arabidopsis Col-0 accession. Furthermore, there is more growth of *Xanthomonas campestris* pv. *campestris* expressing *XopAC* in the *pbl2* background. This suggests that the RLCK, PBL2 is required for the XopAC-triggered immunity [37]. These observations of various pathogen effectors targeting RLCKs are in line with the presumed importance of RLCKs in defense signaling. However, although avrPphB cleaves multiple PBLs/RLCKs [34], only PBS1 cleavage is recognized by RPS5 [27,51]. We report here that PBL1 and BIK1, but neither PBL2 nor PBS1, are required for MAMP/DAMP-induced calcium signaling (Figure 5). This raises the question of “why avrPphB would target PBS1” if PBS1 is not important for MAMP-induced calcium signaling. In fact, there is no evidence so far for any importance of PBS1 in pathogen resistance. As proposed by Zhang *et al.* [46], one idea is that PBS1 may be a “decoy” [52] evolved to recognize perturbation of the real targets of the pathogen effectors. In this case, PBL1 and BIK1 would be such real avrPphB targets, which are relevant for cross-phosphorylation of the receptor complex components and the subsequent triggering of calcium and downstream defense signaling.

## Conclusion

In summary, we showed the requirement for the two RLCKs, PBL1 and BIK1, in MAMP/DAMP-induced calcium signaling, and speculated on possible genotype variations that may differentially contribute to downstream signaling events. There are many more RLCK genes in the genome (Additional file 1: Figure S3) and it remains to be investigated whether these also affect early calcium signal transduction – perhaps in dependence on the type of MAMP/DAMP ligands. The large number of available RLCKs is presumably mirrored by an even wider repertoire of their downstream substrates. Besides phosphorylating receptor complex components, BIK1 was recently shown to target the NADPH oxidase, RBOHD, to control oxidative burst in a calcium-independent manner [36,53]. Thus, a future challenge would be to identify the substrates of these various RLCKs and elucidate their role in cellular signaling.

## Methods

### Plant lines and cultivation conditions

The *Arabidopsis thaliana* lines pMAQ2 in Col-0 background and HVA1 in C24 background were obtained from M. and H. Knight [25]. These lines express the apoaequorin gene under the control of the cauliflower mosaic virus 35S promoter. In the case of HVA1, the aequorin is targeted as a pyrophosphatase (H + −PPase)-apoaequorin fusion protein to the cytoplasmic-face of the tonoplast in the so-called vacuolar microdomain (vmd); thus enabling measurements of calcium changes at this vacuolar vicinity. For the backcross shown in Figure 2, the HVA1 (C24) line was first retransformed with pMAQ2 construct to obtain a line expressing additionally cytosolic apoaequorin (designated HVA1-P). This HVA1-P line was then crossed to the *cce5* mutants. T-DNA lines used in this study are listed in the Additional file 1: Table S1). Plants for ROS assays were grown on soil in climate chambers under short day conditions (8 h light, 16 h dark cycles). For calcium and MAPK assays, seeds were surface-sterilized, stratified at 4°C for >2 d and grown in liquid MS under long day conditions (16 h light, 8 h dark cycles) as described [25].

### Calcium measurements

Seed sterilization, growth of seedlings and other experimental set-up for the calcium measurements in a 96-well plate format was performed as described [26].

### ROS, MAPK and growth inhibition assays

Detection of early MAMP-triggered responses such as MAPK activation and reactive oxygen species (ROS) accumulation was performed as described [9]. As a late response to MAMPs, growth inhibition assay was performed as described [26]. Briefly, seedlings were grown vertically on ATS agar plates with or without 1 μM flg22 for 14 days. To distinguish between growth differences due to treatment versus genotype effects, two-way ANOVA was performed on log_2_-transformed root length data (genotype vs. treatment; p < 0.001; R statistical package) [54]. For a more compact and simplified overview, data in Figure 6 were depicted as percent growth inhibition compared to control.

### Transient expression in protoplasts, immunoprecipitation and autophosphorylation

Transient expression in Arabidopsis protoplasts was performed as described [9]. For each sample, 1 ml of protoplasts (~2 x 10^5^ protoplasts ml^−1^) was transformed; of which 300 μl were kept for western blot analysis of protein expression. The remaining 700 μl were used for immunoprecipitation. Proteins were extracted from the transfected protoplasts as described except that the extraction buffer was supplemented with 1% Triton X-100 [55]. The proteins were incubated with anti-HA (Covance) and protein-G-sepharose (for at least 2 h, 4°C). Washing of the beads was performed as in Lee et al. [55], with centrifugation in between washes to pellet the sepharose beads. Finally, sepharose beads with the immunoprecipitated proteins were resuspended in 20 μl of kinase buffer (20 mM Hepes pH 7.5, 15 mM MgCl_2_, 5 mM EGTA, 1 mM DTT); 5 μl was kept for a western blot to confirm recovery of the HA-tagged proteins. γ^32^P-ATP (3000 Ci mmol^−1^) (0.1 μl) was added to the remaining 15 μl and incubated at 30°C for 1 h to initiate autophosphorylation. Five μl of 4xSDS-loading buffer was added to the beads, incubated at 95°C for 5 min, and 12 μl loaded on a 10% SDS-PAGE. After electrophoresis, the gel was dried, exposed overnight and analyzed by Phosphorimaging.

### Mapping

An F2 population was generated from the cross of *cce5-1* (C24) with the Arabidopsis ecotype Landsberg *erecta* (L*er*-0). F2 plants containing the aequorin transgene (i.e. showing coelenterazine-dependent luminescence) were selected and selfed. DNA was isolated from leaves of F2 plants or from pooled F3 seedlings, and 3 to 5 markers (SNPs, INDELs) of each chromosome were genotyped. Calcium measurements were performed with the F3 seedlings and the segregation of the phenotype used to infer if the corresponding F2 parent is heterozygous or homozygous for the *cce5* mutation.

### Molecular cloning, plant transformation and complementation

For complementation analysis, a genomic fragment covering the PBL1-ORF and 2 kb upstream cis-regulatory region was amplified by PCR using Phusion® Hot Start High-Fidelity DNA polymerase (Thermo Scientific) with primers PBL1-Prom/-STOP and cloned into pENTR™/D-TOPO according to manufacturer’s instructions. Mutation of the N-myristoylation site (G2A) was performed using the QuikChangeII-Kit (Stratagene) with primers PBL1-NMSmut-F/-R according to manufacturer’s instructions. Clones were verified by sequencing and transferred *via* LR reaction into destination vector pGWB1 to obtain *pPBL1*::PBL1 and *pPBL1*::PBL1(G2A). After transfer of the constructs into *Agrobacterium tumefaciens* (GV3101), Arabidopsis *pbl1* mutant plants were transformed by floral-dip transformation. Transgenic plants were selected on hygromycin-containing plates and crossed with *pbl1*-AEQ to introduce the apoaequorin transgene. For transient expression in protoplasts, PBL1- and BIK1-ORFs were amplified from cDNA obtained from Col-0 or the indicated *cce5* alleles by PCR using Phusion® Hot Start High-Fidelity DNA polymerase (Thermo Scientific) with primers PBL1-START/-NoSTOP and BIK1-START/-NoSTOP and cloned into pENTR^TM^/D-TOPO according to manufacturer’s instructions. Mutation of the N-myristoylation site (G2A) was introduced using primers PBL1-STARTmut and BIK1-STARTmut. Clones were verified by sequencing and transferred *via* LR reaction into destination vector pUGW14 to obtain *p35S*::PBL1-3xHA, *p35S*::BIK1-3xHA, *p35S*::PBL1(G2A)-3xHA and *p35S*::BIK1(G2A)-3xHA.

The aequorin-ORF was amplified from plasmid pMAQ2 by PCR using Phusion® Hot Start High-Fidelity DNA polymerase (Thermo Scientific) with primers AEQ-START/-STOP, cloned into pENTR^TM^/D-TOPO according to manufacturer’s instructions, verified by sequencing and transferred *via* LR reaction into destination vector pUB-DEST to obtain *pUBQ10*::AEQ. After transfer of the construct into *Agrobacterium tumefaciens* (GV3101), Arabidopsis Col-0 plants were transformed by floral-dip transformation and transgenic plants were selected by spraying with BASTA® (glufosinat-ammonium; Bayer). All primers used for cloning are listed in the Additional file 1: Table S3).

### Availability of supporting data

All the supporting data are available within the article or as additional files. The phylogenetic tree (Additional file 1: Figure S3) has been deposited in treebase (ID: 16757) and the data will be available at the following URL: http://purl.org/phylo/treebase/phylows/study/TB2:S16757.

## Additional file

Additional file 1: Figure S1. MAPK activation in *cce5* mutants. Reduced MAPK activation in *cce5-1* mutant is seen with flg22 elicitation at low concentrations (10 nM) (A) but not obvious at higher concentrations (100 nM flg22) (B). **Figure S2.** MAMP-induced reactive oxygen species (ROS) accumulation. Reduced ROS accumulation in the *cce5* mutants after elf18 (A) or flg22 (B) treatments. **Figure S3.** Evolutionary relationships of 51 “group VII” RLCKs. **Table S1.** Mutant lines used in this study. **Table S2.** CAPS markers for genotyping the *cce5* mutant alleles. **Table S3.** Primers used for molecular cloning.
