# Supplementary material for: Microbe-associated molecular pattern-induced calcium signaling requires the receptor-like cytoplasmic kinases, PBL1 and BIK1
Source: BMC Plant Biol. 2014 Dec 19;14:374. doi: 10.1186/s12870-014-0374-4 (PMC4279983; doi:10.1186/s12870-014-0374-4)
Supplement: Additional file 1: Figure S1. — MAPK activation in cce5 mutants. Reduced MAPK activation in cce5-1 mutant is seen with flg22 elicitation at low concentrations (10 nM) (A) but not obvious at higher concentrations (100 nM flg22) (B). Figure S2. MAMP-induced reactive oxygen species (ROS) accumulation. Reduced ROS accumulation in the cce5 mutants after elf18 (A) or flg22 (B) treatments. Figure S3. Evolutionary relationships of 51 “group VII” RLCKs. Table S1. Mutant lines used in this study. Table S2. CAPS markers for genotyping the cce5 mutant alleles. Table S3. Primers used for molecular cloning. [file 12870_2014_374_MOESM1_ESM.pdf]

Additional file

Figure S1 – MAPK activation in *cce5* mutants.

Reduced MAPK activation in *cce5-1* mutant is seen with flg22 elicitation at low concentrations (10 nM) (A) but not obvious at higher concentrations (100 nM flg22) (B).

Figure S2 – MAMP-induced reactive oxygen species (ROS) accumulation.

Reduced ROS accumulation in the *cce5* mutants after elf18 (A) or flg22 (B) treatments.

Figure S3 – Evolutionary relationships of 51 "group VII" RLCKs.

The 51 RLCK sequences were based on a classification of "group VII" RLCKs (http://bioinfo.bti.cornell.edu/cgi-bin/itak/index.cgi), with the relevant RLCKs mentioned in this article highlighted in grey. The evolutionary history was inferred using the Neighbor-Joining method [1]. The optimal tree with the sum of branch length = 10.29429802 is shown. The percentage of replicate trees in which the associated taxa clustered together in the bootstrap test (1000 replicates) are shown next to the branches [2]. The tree is drawn to scale, with branch lengths in the same units as those of the evolutionary distances used to infer the phylogenetic tree. The evolutionary distances were computed using the Poisson correction method [3] and are in the units of the number of amino acid substitutions per site. All positions containing gaps and missing data were eliminated from the dataset (Complete deletion option). There were a total of 247 positions in the final dataset. Phylogenetic analyses were conducted in MEGA4 [4].

Table S1 – Mutant lines used in this study.

Table S2 – CAPS markers for genotyping the *cce5* mutant alleles.

Table S3 – Primers used for molecular cloning.

Additional References

1. Saitou N, Nei M: **The Neighbor-Joining Method - a new method for reconstructing phylogenetic trees**. *Molecular Biology and Evolution* 1987, **4**(4):406-425.
2. Felsenstein J: **Confidence-Limits on Phylogenies - an approach using the bootstrap**. *Evolution* 1985, **39**(4):783-791.
3. Zuckerka.E, Pauling L: **Molecules as documents of evolutionary history**. *J Theor Biol* 1965, **8**(2):357-&.
4. Tamura K, Dudley J, Nei M, Kumar S: **MEGA4: Molecular evolutionary genetics analysis (MEGA) software version 4.0**. *Molecular Biology and Evolution* 2007, **24**(8):1596-1599.

Ranf et al. Figure S1

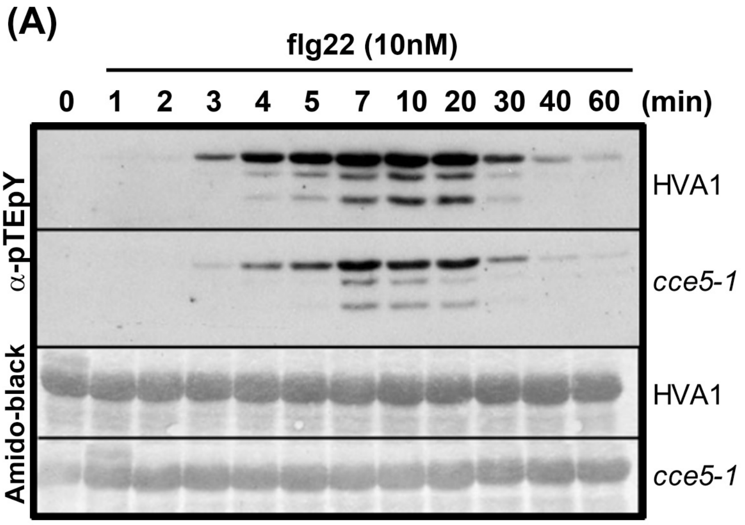

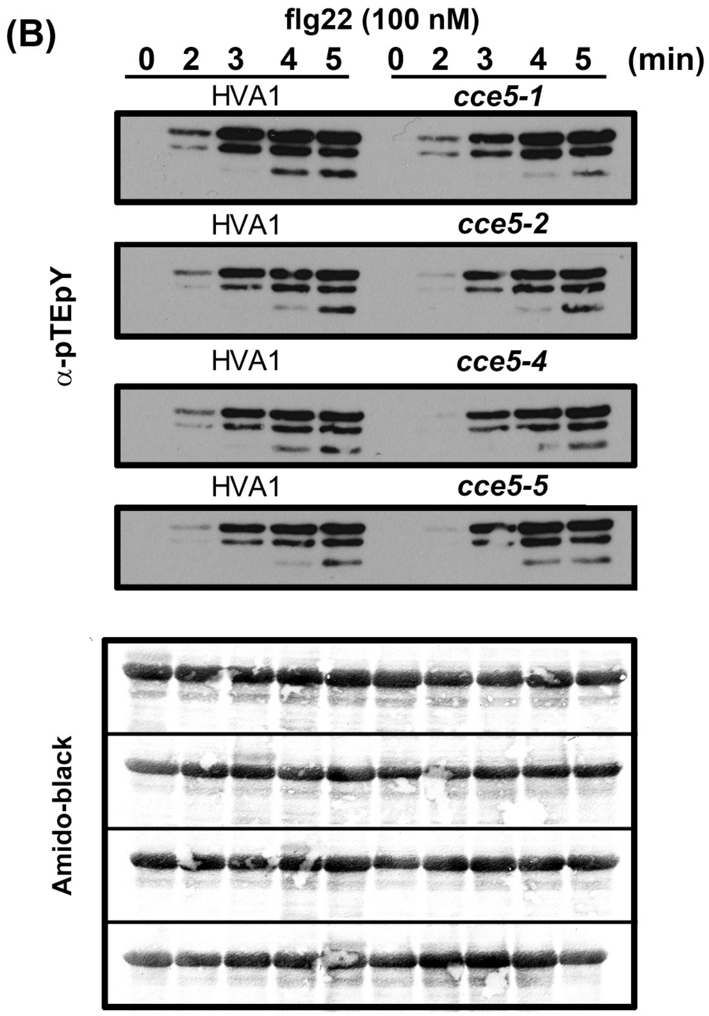

Ranf et al. Figure S2

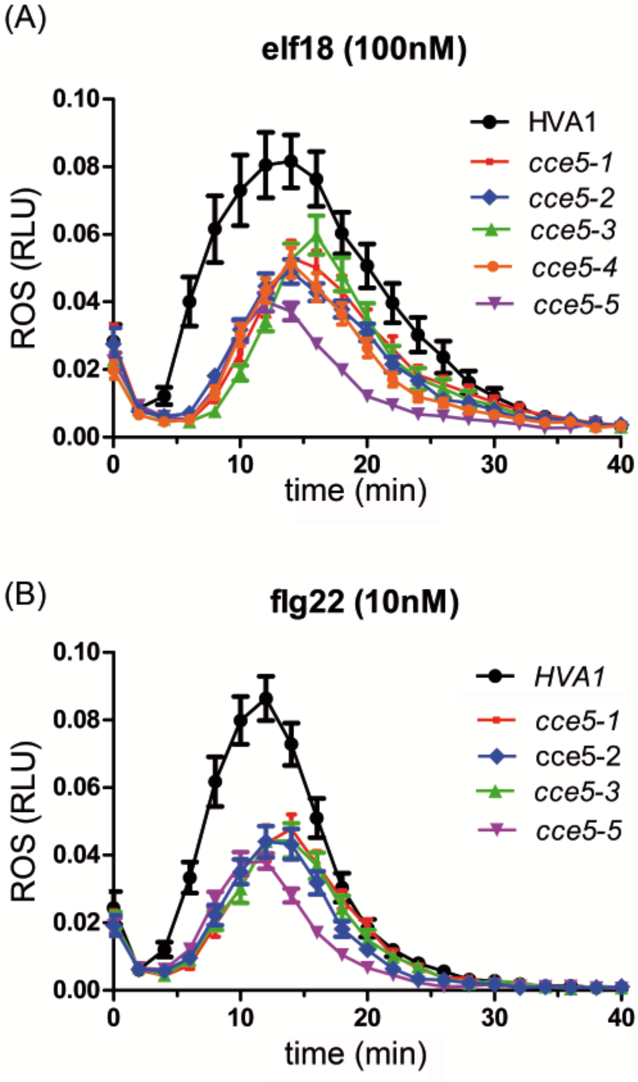

Ranf et al. Figure S3

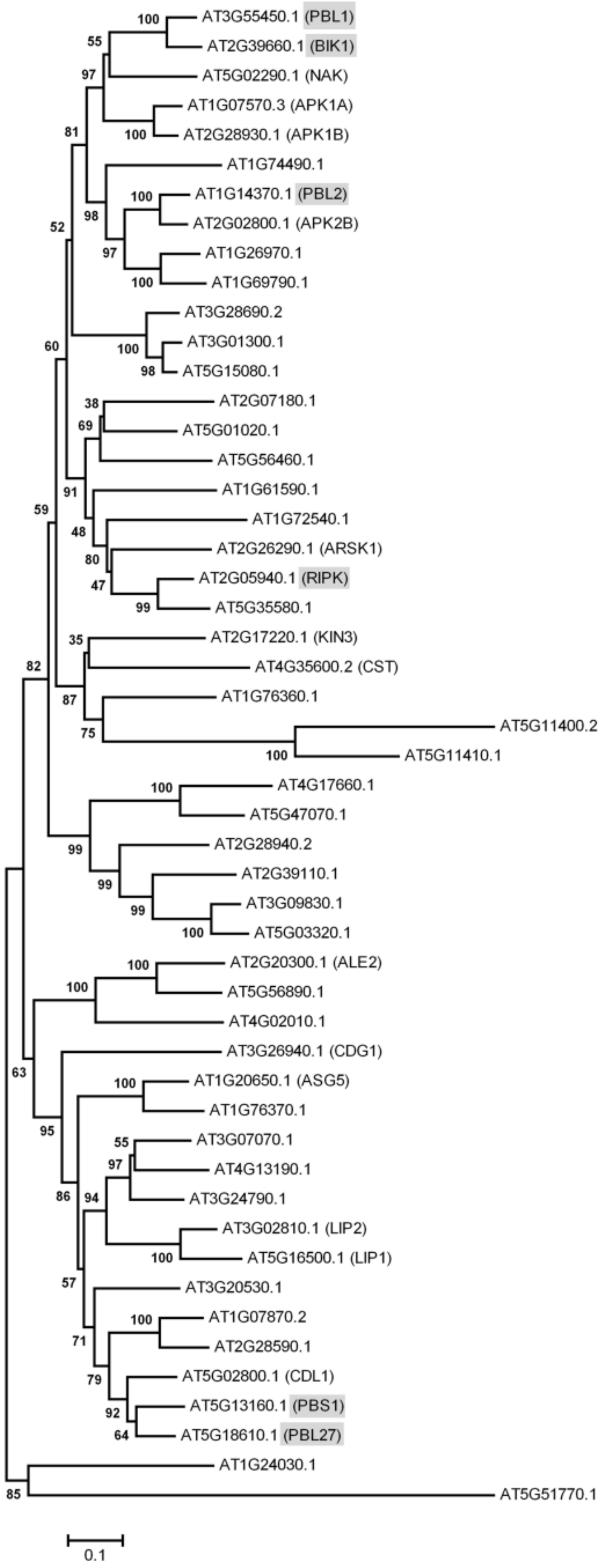

Table S1. Mutant lines used in this study

Mutant lines used are listed below showing the AGI code, identity of insertion line, primers used for genotyping, the aequorin transgenic lines used for crossing and the references (source of the mutants).

mutant	AGI code	insertion line	genotyping primers	aeq. lines	reference	obtained from
bak1-4	At4g33430	SALK_116202	RP: CCGGAGATATTCCTGTTAATGG<br>LP: ACAAGCAATCTTTCGGTTGG	-	Chinchilla *et al.*, 2007	B. Kemmerling
bik1	At2g39660	SALK_005291	RP: GGGTATGGGACATGTAACCGGAAA<br>LP: CAGGTCACTTGAATGCAAGAAGCG	x pMAQ2	Veronese *et al.*, 2006	T. Mengiste
pbl1-1	At3g55450	SAIL_1236_D07	RP: AAGATGTTTGACGCCTTGATG<br>LP: TCCACCCAAAAACAGCATAAG	x pMAQ2<br>x pUBQ-AEQ	Zhang *et al.*, 2010	NASC
pbl2-1 (C12.1)*	AT1G14370	SALK_149140	RP: GGGTCCTTCAGGACTAAGCAC<br>LP: ACCTGTGGCAACATATTCAGG	x pUBQ-AEQ	Zhang *et al.*, 2010	NASC
pbl2-2 (C10.5)*	AT1G14370	GABI_835G07	RP: AAGAAGGTAAAGGCGATGCAC<br>LP: TGGGCAATAAGGATGAAAGTG	x pUBQ-AEQ	-	NASC
pbs1-2 (C3.2)*	AT5G13160	SALK_062464	RP: TATGTACAACCGGAAGATGGC<br>LP: TCCTCTGGTTAAGCTAACGGG	x pUBQ-AEQ	Warren *et al.*, 1999	NASC
serk4-1	At2g13790	SALK_057955	RP: ACGCTCAAGTGGAGTAATGA<br>LP: GCAGCTGAAGAAGACCCAGA	-	Albrecht *et al.*, 2008	S. De Vries
serk5-1	At2g13800	SALK_147275	RP: CTGAAGAAGACCCAGAGG<br>LP: TTGCTTAATGGAAGTGGAGAGA	-	Albrecht *et al.*, 2008	S. De Vries
			SALK_LBb1.3: ATTTTGCCGATTTCGGAAC			
			SAIL_LB1: GCCTTTTCAGAAATGGATAAATAGCCTTGCTTCC			
			GABI_o8409: ATATTGACCATCATACTCATTGC			

*Internal codes for our laboratory collection.

Table S2. CAPS markers for genotyping the *cce5* mutant alleles

cce5 mutant	original name*	SNP	amino acid exchange	CAPS marker: PCR primers	CAPS marker: restriction enzyme (Temp./source)	CAPS marker: fragment sizes for wild type (WT) and mutant (M)
cce5-1	5E3	G465A	G70D	P1: TCAAATCCGTGATCTTTTTGG<br>P2: TCAGGGTTAAGTCGTTTAACAGC	BccI<br>(37°C, NEB)	WT: 438 bp<br>M: 329 + 109 bp
cce5-2	21L8	C546T	R110-	P1: TCAAATCCGTGATCTTTTTGG<br>P2: CATGGACGAGATCAAAGACTCA	TscAI<br>(65°C, Thermo Scientific)	WT: 717 bp<br>M: 458 + 259 bp
cce5-3	63L12	C584T	A97V	P1: AAGGGTCATGGGTACTTTTGG<br>P2: GCCTGCTTGTGAGGTATGGT	BbvI<br>(37°C, NEB)	WT: 182 + 38 bp<br>M: 214 bp
cce5-4	69N19	G941A	Q272-	P1: CAGAGATTAACTATTTGGGGCAGT<br>P2: GCATTTAAGTGGCCTGAAAAA	Cac8I<br>(37°C, NEB)	WT: 307 + 71 bp<br>M: 381 bp
cce5-5	71C11	C1506T	R172Q	P1: CAGCTCATAAGACAGAAGGAGAAA<br>P2: CCATCAGGGTTAAGT	SfcI<br>(37°C, Thermo Scientific)	WT: 762 bp<br>M: 513 + 249 bp

*Internal codes for our laboratory collection.

Table S3. Primers used for molecular cloning

primer name	primer sequence	purpose
PBL1_F	CGGTTGCCTCTACCATCTCA	PCR amplification of genomic fragment from *cce5* alleles for sequencing
PBL1_R	CAAATCGAATATTTCAGGAGCA	PCR amplification of genomic fragment from *cce5* alleles for sequencing
PBL1_F1	TTCTTCAGTGTTTACTTTTTCTCTTTC	sequencing
PBL1_F2	GTGGCTTTGGTTGTGTCTTT	sequencing
PBL1_F3	TTAGCGAGAGACGGTCCAAT	sequencing
PBL1_R1	TCTCGGTGACCTTGAAAACC	sequencing
PBL1-PromF2	TGAAAAATGTCCAACATTACGAA	sequencing
PBL1-PromF3	AACTTATGTCCCATCCCATGA	sequencing
PBL1-Prom	caccGTCCTTTGCTTTTCCAGTCGC	cloning in pENTR-D-TOPO
PBL1-START	caccATGGGTTCTTGTCTCAGTTCTCG	cloning in pENTR-D-TOPO
PBL1-STARTmut	caccATGGcTTCTTGTCTCAGTTCTCG	cloning in pENTR-D-TOPO
PBL1-STOP	CTACAATCCAACGGTTTTTTTGTTTAAACCG	cloning in pENTR-D-TOPO
PBL1-NoSTOP	CAATCCAACGGTTTTTTTGTTTAAACCG	cloning in pENTR-D-TOPO
PBL1-NMSmut-F	CTTCATCATTCTACTAAATGGCTTCTTGTCTCAGTTCTCG	site-directed mutagenesis of NMS

PBL1-NMSmut-R	CGAGAACTGAGACAAGAAGCCATTTAGTAGAATGATGAAG	site-directed mutagenesis of NMS
BIK1-START	caccATGGGTTCTTGCTTCAGTTCTC	cloning in pENTR-D-TOPO
BIK1-STARTmut	caccATGGCTTCTTGCTTCAGTTCTC	cloning in pENTR-D-TOPO
BIK1-STOP	CTACACAAGGTGCCTGCCAAAAG	cloning in pENTR-D-TOPO
BIK1-NoSTOP	CACAAGGTGCCTGCCAAAAG	cloning in pENTR-D-TOPO
AEQ-START	caccATGACCAGCGAACAATATTC	cloning of AEQ-ORF in pENTR-D-TOPO
AEQ-STOP	TTAGGGGACAGCTCCACCGTA	cloning of AEQ-ORF in pENTR-D-TOPO
